# Occupational exposure to glyphosate and risk of lymphoma:results of an Italian multicenter case-control study

**DOI:** 10.1186/s12940-021-00729-8

**Published:** 2021-04-28

**Authors:** Federico Meloni, Giannina Satta, Marina Padoan, Andrea Montagna, Ilaria Pilia, Alessandra Argiolas, Sara Piro, Corrado Magnani, Angela Gambelunghe, Giacomo Muzi, Giovanni Maria Ferri, Luigi Vimercati, Roberta Zanotti, Aldo Scarpa, Mariagrazia Zucca, Sara De Matteis, Marcello Campagna, Lucia Miligi, Pierluigi Cocco

**Affiliations:** 1 Department of Medical Sciences and Public Health, University of Cagliari, Cagliari, Italy; 2 Department of Translational Medicine, Unit of Medical Statistics and Cancer Epidemiology, University of Eastern Piedmont, Novara, Italy; 3 Department of Medicine and Surgery, Occupational Medicine, Respiratory Diseases and Toxicology Section, University of Perugia, Perugia, Italy; 4 Environmental and Occupational Epidemiology Branch - Cancer Risk Factors and Lifestyle Epidemiology Unit, Institute for Cancer Research, Prevention and Clinical Network (ISPRO), Florence, Italy; 5 Interdisciplinary Department of Medicine (DIM), Unit of Occupational Medicine, University of Bari, Bari, Italy; 6 Department of Medicine, Haematology Unit, and Department of Diagnostics and Public Health-Section of Pathology, Verona University Hospital, Verona, Italy

**Keywords:** Glyphosate, Follicular lymphoma, Occupational exposure, Pesticides, Occupational cancer

## Abstract

**Background:**

The International Agency for Research on Cancer (IARC) recently classified glyphosate, the most used herbicide worldwide, as a probable human carcinogen. We inquired into the association between occupational exposure to glyphosate and risk of lymphoma subtypes in a multicenter case-control study conducted in Italy.

**Methods:**

The Italian Gene-Environment Interactions in Lymphoma Etiology (ItGxE) study took place in 2011–17 in six Italian centres. Overall, 867 incident lymphoma cases and 774 controls participated in the study. Based on detailed questionnaire information, occupational experts classified duration, confidence, frequency, and intensity of exposure to glyphosate for each study subject. Using unconditional regression analysis, we modelled risk of major lymphoma subtypes associated with exposure to glyphosate adjusted by age, gender, education, and study centre.

**Results:**

Very few study subjects (2.2%) were classified as ever exposed to glyphosate. Risk of follicular lymphoma (FL) was elevated 7-fold in subjects classified as ever exposed to glyphosate with medium-high confidence, 4.5-fold in association with medium-high cumulative exposure level, 12-fold with medium-high exposure intensity, and 6-fold with exposure for 10 days or more per year. Significant upward trends were detected with all the exposure metrics, but duration. The overall *p*-value for an upward trend with four independent metrics was 1.88 × 10^− 4^. There was no association with risk of lymphoma (any subtype), Non Hodgkin Lymphoma, B-cell lymphoma, or the major lymphoma subtypes other than FL.

**Conclusions:**

Our findings provide limited support to the IARC decision to classify glyphosate as Group 2A human carcinogen.

## Background

Following its 1974 registration in 160 countries, glyphosate is currently the most widely used herbicide worldwide, with a 12-fold increase in production from 67,000 to 826,000 metric tons from 1995 to 2014, and a 25% share of the global herbicide market [1, 2]. Thanks to its broad spectrum of action, glyphosate use is widespread in agriculture to protect wheat and other grain, orchards and fruit and vegetable crops, as well as in households, urban sites, highways, and railroad tracks, against annual or perennial weeds [3]. In 2017, the International Agency for Research on Cancer (IARC) classified glyphosate as a probable human carcinogen (Group 2A), as a result of its unequivocal evidence of carcinogenicity in experimental animals, the limited evidence from epidemiological studies, and the robust evidence of human pertinence of the carcinogenic mechanisms identified in cell cultures [4]. The IARC Working Group observed that mechanistic studies documented two out of 10 characteristics of experimental carcinogens as an effect of glyphosate, namely genotoxicity and induction of oxidative stress [4]. Besides, four metanalyses of case-control studies conducted in the U.S.A. and Europe consistently showed a moderate increase in risk of non-Hodgkin lymphoma (NHL) [5–8] and of the two major lymphoma subtypes, chronic lymphocytic leukaemia (CLL) and diffuse large B cell lymphoma (DLBCL) [7], particularly among subjects who reported using it two or more days per year. The IARC decision prompted a vigorous international debate, fed in part by the different interpretation of the same findings [9], and in part by differences in the main scope of regulatory agencies, and their classification criteria in respect to the IARC hazard assessment.

We explored risk of lymphoma subtypes following occupational exposure to glyphosate in a case-control study, conducted in 2011–2017 in six Italian centres.

## Methods

The “Gene-environment interactions in lymphoma etiology” (ItGxE) multicentre case-control study took place in 2011–2017 in six Italian centres, namely Perugia, Florence, Novara, Verona, Cagliari and Nuoro in Sardinia, and Bari and Taranto in Apulia. The study protocol included a questionnaire interview to the incident cases of lymphoma (any subtype) and to 2:1 age and gender frequency matched controls. In most centres, controls were hospitalized, cancer free patients from surgery wards, eye care departments, or hematology outpatients or patients suffering from trauma injuries, gastrointestinal disorders, cardiovascular disorders. Diagnoses ineligible for selection as hospital controls included malignant neoplasms (any), AIDS, autoimmune diseases, allergic diseases, viral hepatitis, organ transplants, and pre-neoplastic hematologic diseases (monoclonal gammopathy of undetermined significance, bone marrow aplasia, myelodisplastic syndrome).

In the Cagliari and Nuoro centers, controls were a random sample of the general population 2:1 frequency matched to the incident case by 5-year age and gender groups. The overall refusal rate was 7.4% among the cases and 38.4% among the controls; it was 41.1% among the population controls, and 37.1% among the hospital controls. Overall, 867 cases (500 males and 367 females) and 774 controls (428 males and 346 females) participated in the study. Trained interviewers conducted in person interviews at the hospital or at the residence of study subjects, using a modified version of the EpiLymph questionnaire [10], designed to gather information on a number of variables, including socio-demographic data, and a lifetime occupational history. For each job lasting 1 year or more, specific questions asked for a short description of the employer’s trade, the daily tasks, the machines and tools used, and a self-report about exposures of interest for the study. A set of 14 additional job modules, including one dedicated to gardeners and farmers, addressed further details on exposures deemed of interest based on previous epidemiological findings. The detailed information acquired through the specific job module for gardeners and farmers included type of crops, the size of each crop field, type of phytopathology treated, type of pesticides used, days/year of treatment, spraying tools, use of personal protective equipment, and post-treatment re-entry in the fields before the expiry of the respect time. Based on the above-described information, and with the support of a “*crop-exposure*” matrix [11], occupational physicians and industrial hygienists, with expertise in the retrospective assessment of agricultural exposures, assessed exposure to glyphosate using the following semi-quantitative indicators:

- confidence, representing the degree of certainty about whether a given study subject had actually been exposed. Three increasing level of confidence were defined depending on 1. a summary evaluation of the probability of exposure (0 = unexposed; 1 = possible, but not probable; 2 = probable; and 3 = certain); and 2. the proportion of exposed among workers performing the same tasks (1 = ≤40%, 2 = 40–90%; 3 = ≥90%), in relation to availability of alternate herbicides, and/or local market data;
- intensity of exposure, based on the exposure circumstances (personal preparation of the pesticide mix, use of a shoulder pump or a tractor with or without a cabin, size of the surface to be treated, re-entry after treatment) and the use of personal protective equipment. Semi- quantitative exposure estimates were obtained from the publicly available EUROPOEM spreadsheet [12] (https://english.ctgb.nl/documents/assessment-framework-ppp/2016/10/27/ calculation-model-europoem-ii), and classified in a four-step scale (0 = unexposed; 1 = low; 2 = medium; 3 = high). Intensity of exposure at the individual level was the highest along the work history of the study subject.
- frequency of exposure, in terms of days/year of use of the herbicide, as reported in the questionnaire and/or estimated based on the type of treatment whether curative or preventive, and the size of the surface to be treated (low frequency = ≤5 days/year; medium frequency = 5–10 days/year; high frequency = ≥11 days/year).

A score of cumulative exposure to glyphosate was then calculated as it follows [13]:

$$ {C}_i=\Sigma\ {\left({\mathrm{y}}_j\mathrm{x}\ {\mathrm{f}}_j/3\right)}^{\mathrm{x}j} $$

where,

C_*i*_ = score of cumulative exposure for the *i*th study subject;

*j* = *j*th job entry on the work history of the *i*th study subject;

y_*j*_ = duration of exposure (in years) of the *j*th job entry;

x_*j*_ = level of exposure intensity in the *j*th job entry;

f_*j*_ = level of frequency of exposure in the *j*th job entry.

Duration of exposure was approximately calculated from 1974, year of introduction of glyphosate in the market, onwards.

Lymphoma is a complex array of different neoplastic diseases that develop from the lymphatic tissue, which classification changed multiple times in the last 5 decades. Non-Hodgkin lymphoma (NHL), includes all lymphoma subtypes, independent on whether originating from B or T lymphocytes, and it excludes chronic lymphocytic leukemia (CLL) and multiple myeloma (MM). Although clinically obsolete, the NHL definition keeps being used in clinical settings to classify patients whose pathological diagnosis and immuno-histochemistry is not available yet, and by epidemiologists to preserve the possibility of making comparisons with past results. We classified lymphoma according to the 2008 update of the WHO classification of lymphoma [14], which relies on morphology, immunophenotype, molecular biology, genetics, and clinical presentation and course of the disease, and it includes all the lymphoma subtypes originating from B-lymphocytes, including CLL and MM, among the group of B-cell lymphoma (BCL). T-cell lymphomas, and Hodgkin lymphoma (HL) are separately classified.

### Statistical methods

We used unconditional logistic regression models to assess risk of lymphoma (all subtypes), the NHL and BCL groups, HL, and the major BCL subtypes, including diffuse large B cell lymphoma (DLBCL), chronic lymphocytic leukaemia (CLL), follicular lymphoma (FL), and multiple myeloma (MM), associated with ever exposure (including all categories of confidence, intensity, frequency and duration of exposure), and with categories of confidence, duration, intensity, and frequency of exposure to glyphosate, as well as with cumulative exposure to glyphosate. The following covariates were included in the regression models: age (continuous), gender, study center, and education. Education level was used as a surrogate for social class-related risk factors, and it was categorized as primary school, middle school, or vocational studies (≤8 years), up to high school graduation (9–12 years), and academic and university studies up to achieving degree (≥ 13 years).

The measure of association was the Odds Ratio (OR) and its 95% confidence interval (95% CI). We tested linear trends by the exposure metrics with the Wald test for trend (β/*se*_β_), after continuous transformation of all the categorical variables in the regression model*.* We also applied the Fisher test for combined probabilities to calculate the chance probability of observing a positive trend with four different metrics bearing upon the same overall hypothesis, namely confidence, duration, frequency, and intensity of exposure, assumed as reciprocally independent [15]. We used the Cochran’s Q test to detect heterogeneity in risk across lymphoma subtypes. All the analyses were conducted with SPSS® version 20.0.

Local Ethics Committees approved the study protocol in all the participating centers. Informed consent was obtained from each participant.

## Results

Table 1 shows selected characteristics of the study population and the distribution of cases and controls by participating centre. The male/female ratio among the cases was 1.4:1, consistently with the expectation. The mean age of study participants was 55.1 years (standard deviation [*sd*] 15.6), and it did not vary by case-control status; males were slightly older (56.6 years old, *sd* 14.6) than females (53.1 years, *sd* 16.3) (*t* = 4.57, *p* = 5.12 × 10^− 6^). Cases were slightly less educated than the controls. Overall, one third of the study population had a high school degree, and a little less than two thirds had at least a middle school diploma. Only 36 study subjects (2.2%) had ever been exposed to glyphosate, and 15 were classified in the medium or high categories of confidence of exposure. Therefore, we combined the medium and high categories for the purposes of analysis.

**Table 1:** Selected characteristics of the study population by case-control status and overall

***Gender***	***Cases*** ***N %***	***Controls*** ***N %***	***Total*** ***N %***
Total	867 (100)	774 (100)	1641 (100)
Males	500 (57.6)	428 (55.3)	928 (56.6)
Females	367 (42.4)	346 (44.7)	713 (43.4)
M/F ratio	1.4/1	–	–
***Age***	***Mean (sd)***	***Mean (sd)***	***Mean (sd)***
Total	55.2 (15.9)	55.1 (15.4)	55.1 (15.6)
Males	56.7 (14.2)	56.9 (14.4)	56.6 (14.6)
Females	52.7 (16.3)	54.4 (16.9)	53.1 (16.3)
***Study center***	***Cases*** ***N %***	***Controls*** ***N %***	***Total*** ***N %***
Bari/Taranto	210 (24.2)	108 (14.0)	318 (19.4)
Cagliari/Nuoro	207 (23.1)	227 (29.3)	434 (26.4)
Firenze	228 (26.3)	188 (24.3)	416 (25.3)
Novara	102 (11.8)	85 (11.0)	187 (11.4)
Perugia	90 (10.4)	98 (12.7)	188 (11.5)
Verona	30 (3.3)	68 (8.8)	98 (6.0)
***Education***	***Cases*** ***N %***	***Controls*** ***N %***	***Total*** ***N %***
Primary school	167 (19.3)	107 (13.8)	274 (16.7)
Middle school	262 (30.2)	231 (29.8)	493 (30.0)
High school	265 (30.6)	284 (36.7)	549 (33.4)
University	123 (14.2)	122 (15.8)	245 (14.9)
Other	35 (4.0)	19 (2.5)	54 (3.4)
Missing	15 (1.7)	11 (1.4)	26 (1.6)
***Exposure to glyphosate***	***Cases*** ***N %***	***Controls*** ***N %***	***Total*** ***N %***
Unexposed	846 (97.6)	759 (98.1)	1605 (97.8)
Ever exposed	21 (2.4)	15 (1.9)	36 (2.2)
Low confidence	12 (1.4)	9 (1.2)	21 (1.3)
Medium-high confidence	9 (1.0)	6 (0.8)	15 (0.9)

Table 2 breaks down cases by diagnosis. Immunohistochemistry was available for 637/867 cases (73.5%) to characterize the subtype. For the rest of the cases, 175 had a clinical diagnosis of NHL, 22 a diagnosis of BCL, and 33 a diagnosis of lymphoma not otherwise specified.

**Table 2:** Lymphoma cases by diagnostic group and subtypes

***Diagnostic group/subtype of lymphoma***	*N (%)*
Diffuse large B-cell lymphoma	99 (11.4)
Follicular Lymphoma	86 (9.9)
Chronic lymphocytic leukemia and small cell lymphocytic lymphoma	83 (9.6)
Mantle cell lymphoma	20 (2.3)
Marginal zone and MALT lymphoma	35 (4.0)
Multiple myeloma	95 (11.0)
B-cell lymphoma, other subtypes	16 (1.8)
B-cell lymphoma, nos	22 (2.5)
T-cell lymphoma	23 (2.7)
Hodgkin lymphoma	180 (20.8)
Non Hodgkin Lymphoma nos	175 (20.2)
Lymphoma nos	33 (3.8)
All diagnoses	867 (100.0)

Table 3 shows risk of lymphoma (any subtype), NHL, BCL, BCL subtypes, and HL associated with ever exposure to glyphosate. Overall, results would not support an association with lymphoma in general, nor with any specific group or subtype, but FL, which showed a 3.7-fold excess (95% CI 1.06–12.79). The exposure prevalence was too low, and the study size therefore insufficient to detect the weak, non-significant association with BCL risk and MM risk. No heterogeneity was detected across lymphoma subtypes (Cochran’s Q = 2.64; df = 4; *p* = 0.619). After excluding the FL cases, BCL risk associated with ever exposure to glyphosate was moderately increased (OR = 1.3, 95% CI 0.53–3.08), but there was no association with medium-high probability of exposure (OR = 0.7, 95% CI 0.15–2.97) (not shown in the tables).

**Table 3:** Risk of lymphoma (any subtype), B cell lymphoma, its major subtypes and Hodgkin lymphoma associated with ever exposure to glyphosate

*Lymphoma group/subtype*	*Unexposed* *Ca/Co OR*	*Ever Exposed* *Ca/Co OR 95%CI*	*Exposed with medium-high confidence* *Ca/Co OR 95%CI*
*Lymphoma (any subtype)*	846/759 1.0	21/15 1.0 0.51–2.05	9/6 1.2 0.40–3.46
*Non Hodgkin Lymphoma*	462/759 1.0	14/15 1.4 0.62–2.94	5/6 1.2 0.35–4.21
*B-cell lymphoma*	443/759 1.0	14/15 1.6 0.73–3.66	6/6 1.6 0.48–5.44
*Diffuse large B cell lymphoma*	98/759 1.0	1/15 0.8 0.10–6.62	0/6 - -
*Follicular lymphoma*	82/759 1.0	4/15 3.7 1.06–12.8	3/6 7.1 1.57–31.9
*Chronic lymphocytic leukemia*	81/759 1.0	2/15 0.6 0.11–3.48	0/6 - -
*Multiple Myeloma*	91/759 1.0	4/15 1.6 0.45–5.88	0/6 - -
*Other B-cell Lymphoma*	75/759 1.0	2/15 1.2 0.26–5.92	0/6 - -
*Hodgkin Lymphoma*	179/759 1.0	1/15 0.3 0.03–2.27	0/6 - -

Risk of lymphoma (any subtype), NHL, and BCL did not change after excluding from the analysis the cases and controls in the low category of confidence of exposure. Instead, FL risk increased up to 7-fold (95% CI 1.57–31.9), while no cases of the other lymphoma subtypes were represented in this group.

Table 4 shows risk in relation to the exposure metrics. Risk of BCL, but not risk of all lymphoma combined, increased by intensity, frequency and confidence of exposure, but not by duration. NHL risk increased slightly by intensity and cumulative, but not duration nor frequency of exposure. Risk of FL was always elevated in the top category of all exposure metrics, although the increase by duration was not linear. BCL risk (OR = 4.0, 95% CI 1.38–11.4), and FL risk (OR = 12.0, 95% CI 2.95–49.0) were highest in association with medium-high exposure intensity. A linear increase in risk was also observed by cumulative exposure for both BCL and FL, but the small study size limited the interpretation of the observed association.

**Table 4:** Risk of lymphoma (any subtype), B cell lymphoma and follicular lymphoma in relation to the glyphosate exposure metrics with reference to the unexposed

Exposure metrics	Lymphoma (any subtype) Ca/Co OR 95% CI	Non Hodgkin lymphoma Ca/Co OR 95% CI	B cell lymphoma Ca/Co OR 95% CI	Follicular lymphoma Ca/Co OR 95% CI
*Duration of exposure*				
*Unexposed*	846/759 1.0 -	462/759 1.0 -	443/759 1.0 -	82/759 1.0 -
*≤ 16 years*	11/7 1.2 0.44–3.11	8/7 1.9 0.67–5.64	7/7 2.5 0.79–7.80	2/7 8.2 1.43–46.7
*≥ 17 years*	10/8 0.9 0.33–2.36	6/8 0.9 0.29–2.78	7/8 1.1 0.38–3.36	2/8 2.1 0.39–11.5
*Intensity of exposure*				
*Low*	4/9 0.3 0.09–1.05	1/6 0.2 0.05–1.52	1/6 0.3 0.05–1.52	0/9 -
*Medium - high*	17/6 2.1 0.80–5.48	13/9 2.2 0.90–5.31	13/9 4.0 1.38–11.4	4/8 12.0 2.95–49.0
*Frequency of exposure*				
*Low*	9/8 0.9 0.33–2.38	8/8 1.6 0.56–4.44	6/8 1.5 0.48–4.75	1/8 1.5 0.15–15.0
*Medium - high*	12/7 1.2 0.44–3.08	6/7 1.1 0.35–3.51	8/7 1.8059–5.33	3/7 6.0 1.40–26.1
*Cumulative exposure*				
*Low*	10/9 0.8 0.33–2.16	7/9 1.2 0.42–3.45	7/9 1.4 0.48–4.31	2/9 3.1 0.53–17.6
*Medium - high*	11/6 1.3 0.46–3.59	7/6 1.5 0.49–4.81	7/6 1.9 0.60–6.01	2/6 4.5 0.82–24.1

The test for trend in risk of B-cell lymphoma was *p* = 0.474 for duration of exposure; *p* = 0.329 for confidence; *p* = 0.501 for frequency; and 0.061 for exposure intensity. That of follicular lymphoma was *p* = 0.121 for duration of exposure; *p* = 0.015 for confidence; *p* = 0.019 for frequency; and *p* = 0.008 for exposure intensity. The Fisher method for combined probability yielded a chance probability of an upward trend in BCL risk of *p* = 0.527, and that of FL was *p* = 1.88 × 10^− 4^.

## Discussion

Our results offer limited support to the hypothesis that medium-high level exposure to glyphosate prolonged for 10 days or more per year might increase risk of follicular lymphoma, but not lymphoma overall, nor the NHL category, other major B cell lymphoma subtypes, or Hodgkin lymphoma. Risk of follicular lymphoma was significantly elevated in the top category of three exposure metrics, namely confidence, frequency, and intensity, but not duration of exposure.

Neither diffuse large B-cell lymphoma, nor chronic lymphocytic leukemia, nor Hodgkin lymphoma showed any association with exposure to glyphosate. Exposure to glyphosate was rare in our study (ever exposed 2.2%; exposed with medium-high confidence 0.9%), but it was even rarer in a previous Italian multicenter case-control study on NHL (ever exposed 0.3%) [16], and in the European EpiLymph study (ever exposed 0.1%) [13].

The results of the large prospective US Agricultural Health Study (AHS) showed a 2.6-fold (95% CI 0.7–9.4) increase in risk of multiple myeloma, after multiple adjustments, but not non-Hodgkin lymphoma [17]. Although not significant, the finding corroborated those from previous case-control studies conducted in Iowa and Minnesota (OR = 1.7, 95% CI 0.8–3.6) [18], in Iowa [19], in Canada [20], and in France (OR = 2.4, 95% CI 0.8–7.3) [21]. However, the analysis of risk of monoclonal gammopathy of undetermined significance (MGUS), a condition known for its tendency to evolve towards multiple myeloma, did not support those findings [22]. We did observe a 60% increase in risk of multiple myeloma associated with ever exposure to glyphosate, but our study did not achieve enough statistical power to test the association. On the other hand, in a Canadian study, use of glyphosate for two or more days/year was associated with a 2.12-fold increase in risk of NHL (95% CI 1.20–3.73), with reference to users for 1–2 days/year or unexposed [23]. Risk of NHL was not elevated in the French study mentioned above [21], while in the European multicenter EpiLymph case-control study four cases and two controls had been ever exposed to glyphosate (OR = 3.1, 95% CI 0.6–17.1) [13]. Finally, risk for Hodgkin lymphoma was elevated in the French case-control study (OR = 1.7, 95% CI 0.6–5.0) [21], but not in a multicentre Canadian case-control study (OR = 1.14, 95% CI 0.74–1.76) [24].

In most studies, NHL was the definition of the disease entity. Such definition encompasses an array of diseases each with their own causal models and biomolecular features, with different prognosis, and more or less responsive to the various treatments [25]. When looking at our cases fitting the definition of NHL, we did not find an association with exposure to glyphosate. Among lymphoma subtypes, two small size Swedish studies reported an association with hairy cell leukemia, a rare B cell lymphoma subtype [26, 27]. A larger study confirmed a 3.04-fold excess risk (95% CI 1.08–8.52) of hairy cell leukemia, based on 8 cases, and a more than two-fold increase in risk of all lymphomas (OR = 2.36, 95% CI 1.04–5.37) associated with exposure to glyphosate lasting 10 or more days/year [28]. Risk was particularly elevated for chronic lymphocytic leukemia and small lymphocytic lymphoma combined (OR = 3.35, 95% CI 1.42–7.89).

The varying prevalence of follicular lymphoma in the case series so far investigated might account for the contradictory findings across the epidemiological studies. However, the pooled analysis of three agricultural cohorts co-ordinated by the International Agency for research on Cancer (the AGRICOH Consortium), did not find an excess risk of follicular lymphoma in association with ever exposure to glyphosate [29]. Instead, an excess risk of DLBCL was observed after stratification by DDT exposure. In this study, the exposure assessment differed across cohorts, and it was based mainly on self-reports, which were combined with crop-exposure matrices in the French and Norwegian cohort [29]. A non-significant 40% excess risk of follicular lymphoma was observed among the ever exposed to glyphosate in the French case-control study [21], based on 3 exposed cases and 24 exposed controls. In the French study, which data set was part of the above mentioned AGRICOH study, exposure was classified as *possible or definite*, depending on whether just reported by the study participants or also confirmed by experts, although duration was also mentioned as an exposure variable [21]. However, a more detailed analysis was not conducted because of the small number of FL cases (*N* = 50) and the low prevalence of exposure.

In our study, the prevalence of exposure to glyphosate was low, although it was higher than in previous studies [13, 16]. Ever exposed in our study accounted for about 2% of the study subjects, which is plausible due to the progressive downsizing of the agricultural workforce along the last decades, the fact that crops requiring herbicide treatments are a fraction of the overall agricultural products, and the limited extension of crops requiring herbicide treatment in some study areas.

Therefore, our study suffered from low statistical power to detect associations and from a higher probability of chance findings due to small numbers, which are major interpretative limitations. Besides, we only assessed exposure to glyphosate, but not to any other pesticides. Organophosphorous insecticides and lindane have been associated with an increase in risk of lymphoma [13, 30], and IARC considers lindane as a Group 1 human carcinogen [30]. However, agricultural uses of lindane have been discontinued long ago. If collinearity would exist between use of organophosphorous insecticides and that of glyphosate, our results might have been biased. However, such occurrence seems unlikely, though possible, due to the diversity between crops requiring herbicide treatments (mainly wheat, cereals and grains, ornamental plants, lawns, and gardens) and those requiring insecticide treatments (mainly orchards, and olive trees).

Further reasons for concern in interpreting our results are related to the different participation rate among the cases and the controls. Although the refusal rate in our study was similar to other case-control studies, we do not have details on whether refusals differed from the participants in respect to the adjusting covariates. We relied on multivariate analysis to adjust for confounders, but we cannot be sure about whether and to what extent selection bias and residual confounding might have affected our results. However, information about work circumstances, type of crops, type of treatments, and occasionally type of pesticides used, was self reported by study participants; such information was filtered through the expertise of industrial hygienists and occupational physicians, to come up to the exposure metrics. Therefore, self reported information contributed, but it was not the exclusive source of information. Also, at the time of conducting the study glyphosate had not been at the center of media attention as yet. We are confident that there was no reason for differential reporting about exposure by case-control status, and that misclassification of exposure might have been equally distributed between cases and controls.

## Conclusions

Our findings provide limited support to the IARC decision to classify glyphosate as a probable human carcinogen (Group 2A), with specific lymphoma subtypes as the target. Further larger size studies, supported by a retrospective exposure assessment at least as accurate as the one we conducted, are warranted to strengthen the hypothesis.

## Data Availability

The datasets used and/or analysed during the current study are available from the corresponding author on reasonable request.

## Abbreviations

BCL: B-cell lymphoma
CLL: Chronic lymphocytic leukaemia
DLBCL: Diffuse large B cell lymphoma
FL: Follicular lymphoma
HL: Hodgkin lymphoma
ItGxE: Italian Gene-Environment Interactions in Lymphoma Etiology
NHL: Non-Hodgkin lymphoma
MGUS: Monoclonal gammopathy of undetermined significance
MM: Multiple myeloma
OR: Odds Ratio
*sd*: standard deviation
IARC: The International Agency for Research on Cancer
AHS: US Agricultural Health Study
95% CI: 95% confidence interval
